# The burden of surgical site infection and associated factors among patients admitted to the surgical ward in resource-limited countries: an institutional-based cross-sectional study

**DOI:** 10.3389/fsurg.2025.1571033

**Published:** 2025-08-28

**Authors:** Simachew Zewdu, Abel Daniel, Amene Abebe, Zinabu Abraham, Hailu Elias, Adamu Belete

**Affiliations:** 1 Department of Surgery, School of Medicine, Wolaita Sodo University, Sodo, Ethiopia; 2 School of Public Health, Wolaita Sodo University, Sodo, Ethiopia; 3 Department of Orthopedic and Trauma Surgery, Gondar University, Gondar, Ethiopia

**Keywords:** surgical site infection, surgical ward, burden, resource-limited setup, factors

## Abstract

**Background:**

Surgical site infections (SSIs) are a leading cause of morbidity and mortality worldwide. Particularly, in low- and middle-income countries (LMICs), they are the most prevalent kind of healthcare-associated infection (HAI), and they play a role in the emergence of antibiotic resistance, which can result in serious illnesses. Therefore, this study aims to ascertain the burden and association of surgical site infection among patients on the surgical ward in resource-limited surgical setups.

**Method:**

An institutional-based cross-sectional study was conducted in Wolaita Sodo University Comprehensive Specialized Hospital from March 1, 2022 to July 30, 2023. A systematic random sampling method was employed. Data management and statistical analysis were performed using SPSS version 25. An adjusted odds ratio (AOR) with 95% confidence interval was used to measure the association between dependent and independent variables. A *p*-value < 0.05 was used to determine the level of significance.

**Result:**

This study included a total of 309 patients, of whom 198 (64.1%) were males. The average age of the participants was 42, and participants more than 42 years’ old totaled 156 (50.5%); the type of residence was found to be rural for 236 patients (84.6%). The magnitude of surgical site infection was calculated to be 29.1%. Predisposing factors for surgical site infection included male sex (AOR −4.9; 95%; 2.0–11.3), drainage use (AOR −4.46; 95%; 1.9–10.3), and abdominal surgery (AOR−4.3; 95%; 1.3–14.1), whereas protective factors included younger female sex, elective surgery, and a surgery duration of less than 2 h.

## Background

Surgical site infections (SSIs) are a preventable consequence of surgery that pose a serious risk to public health, affecting not only patients but also the financial and human resources of the medical field (1). It' is one of the growing concerns in nosocomial infections. This issue is made worse by the steady rise in antibiotic resistance, the growing number of therapies, and the increasingly complex type of patients as a result of their comorbidities (2). The World Health Organization (WHO) states that there are 1.2–23.6 SSIs for every 100 surgical procedures worldwide (3), and its cumulative incidence ranged from 2.5% to 30.9% in Africa (4). According to reports, SSIs account for almost one-third of postoperative fatalities globally (5), and it annually endangers the lives of millions of people and fuels the development of antibiotic resistance (6).

An SSI is characterized by a surgical wound that exhibits localized infection symptoms and, in more severe cases, systemic symptoms such as fever or leukocytosis (7). It can develop within 30 days following a surgical procedure (or within 90 days for surgeries involving the implantation of prosthetic material) at the incision site and/or deeper underlying tissue spaces and organs. SSIs make up roughly 38% of all surgically related nosocomial infections (8).

According to a comprehensive assessment conducted in 2020, SSIs have a significant impact on LMICs, accounting for 38% of all deaths. LMICs saw a greater rate of SSIs than HICs in Europe, with the patient bearing the majority of the costs when medical expenses incurred as a result of the incident surpassed 10% of the household's yearly income (9–11). The SSI rate covered between 2% and 5% European countries (12), while the pooled prevalence rate in LMICs was 11.2% (13). In Sub-Saharan Africa, the rate of impact of SSIs ranged from 6.8% to 26% with a predominance in general surgery (14), and other studies showed that its rate of prevalence was about 2.5–30.9% (4, 15–17). In Tanzania, 22% of patients developed SSIs after open urologic surgery (18), and studies reported SSI rates of 19.4% and 24% in the district and tertiary hospitals of Tanzania, respectively (19). The rate was 16.4% in Uganda (20) and 13.0–22.05% in Nigeria (21). In Ethiopia, the pooled prevalence rate of SSIs was 12.3% (1) as reported in one study and 25.22% (22) as reported in another study.

An SSI is associated with a number of risk factors, including identifiable intrinsic and extrinsic factors. Modifiable inherent risk factors include diabetes, respiratory conditions and other preexisting illness (19, 23, 24), smoking, steroid use, alcoholism, obesity, immunocompromised individuals, albumin levels <3.5 mg/dl, and anemia and bilirubin levels >1.0 mg/dl. Age, type of surgery, and recent radiation therapy (25, 26) are non–modifiable risk factors. Clean wounds are usually closed, are free of infection, and show no symptoms of inflammation. Clean-contaminated wounds are wounds that have a low level of contamination, while contaminated wounds have a high level of contamination and typically result from a breach in sterile techniques or leakage from the gastrointestinal tract. Dirty wounds are grossly infected and usually occur because of an inadequate treatment of traumatic wounds, gross purulence, and evident infections (1, 20, 27, 28). An American Society of Anesthesiologists (ASA) Score III or IV (27, 29), non-use of prophylactic antibiotics, (30) presence of hypovolemia (19, 31), longer duration of operation (19, 23, 30), and longer preoperative (23, 30) and postoperative hospital stay (23, 28) are common determinants of SSIs across studies. SSIs continue to be a significant contributor to hospital-acquired infections even with advances in operating room procedures, equipment sterilization techniques, and surgical techniques and varies from setup to setup. Therefore, this study aims to ascertain the burden and associated factors of SSIs in peripheral teaching hospitals in resource-limited settings.

## Method

A retrospective institutional-based cross-sectional study was conducted between August 1 and 30, 2023, at Wolaita Sodo University Comprehensive Specialized Hospital (WSUCSH) located in Wolaita Zone, Ethiopia, among patients who were operated upon and admitted to the surgical ward March 01, 2022 to July 30, 2023. The hospital serves as a teaching institution for health science students and surgical residents and provides 24-hour comprehensive services for more than 6 million people with different demographic and socioeconomic backgrounds from in and around the southern part of the country. It offers general surgery, internal medicine, neurology, orthopedics, neurosurgery, obstetrics and gynecology, pediatrics, radiology, dermatology, pathology, oncology, anesthesiology, and neonatal care specialty services in the respective departments for the entire population of southern Ethiopia.

### Source and study population

All patients were operated upon and admitted to the WSUCSH surgery department during the period of the study in both private and public wards.

### Inclusion criteria

Adult patients who were operated upon and admitted to the surgery department and had a complete medical record.

### Exclusion criteria

Patients with lost or incomplete charts or those who underwent obstetric or gynecological surgery.

### Sample size determination and sampling technique

The sample size for the first objective was calculated using a single population proportion formula considering p 24.6% taken from a study done in Hawassa, with 95% CI and a margin of error of 5%. This gave a sample size of 285. Then, if a 10% non-response rate was added, the final sample size became 314.

$$n=\frac{{\left(\frac{Z\alpha }{2}\right)}^{2}*p*(1-p)}{d^{2}}\;\;n=(1.96{)}^{2}(0.246)(0.754)/(0.05{)}^{2}$$

*n* = 285

where

*n* = desired sample sizes

Z*α*/2 == critical value at 95% CI, which equals to 1.96 P = proportion of SSI at Hawasa 0.246,.

d = margin of error (0.05).

The sample size for the second objective was calculated with Open Epi using an assumption of power of 80%, 95% CI, and an exposed/unexposed ratio of 1:1. Therefore, for the maximum sample size is the sample size required for the first objective. Then the final sample size becomes 314. A systematic random sampling method was employed to select the study subjects.

### Dependent variable: surgical site infection

#### Data collection tools and methods

Data were collected using a validated, pretested, and structured data extraction checklist adopted from relevant literature and modified to the study variables. First, the operation theatre and admission records were reviewed to prepare lists of patients operated upon and admitted to the surgical ward between August 1, 2023 and August 30, 2023. Then, data were extracted from the medical registrations of patients taken from the examination room on arrival, operating room records, postsurgical evaluation and monitoring sheets, and intensive care and discharge records. Data were collected by trained data collectors and supervisors through a review of the medical records of the patients.

#### Data quality management

A pretested validated structured data collection tool prepared in simple English after a review of related literature was used to ensure data quality. One day of training was given to data collectors and supervisors on the purpose of the study, the contents of the data collection tool, where to find the records, and how to extract the required data from the medical records and record data appropriately. A pretest was conducted on 5% of the sample size before the actual data collection period to check for the reliability and validity of the data collection tool. The questionnaires were reviewed and checked for completeness, accuracy, and consistency by the principal investigator and amended accordingly based on the pretest results. Supervisors and the principal investigator carefully checked the collected data for completeness, accuracy, and consistency daily. Two individuals performed double data entry to minimize errors.

#### Data processing and analysis

The collected data were validated for completeness and accuracy and categorized, coded, and analyzed using Statistical Package for Social Sciences (SPSS) software version 25.0. Descriptive statistics were used for frequency, mean/median, standard deviation, and percentage. The results were summarized using graphs, charts, and tables. The interpretations of data using summaries were done according to the main finding in the study objective. Thus, variables having a *P*-value < 0.25 at a 95% confidence interval (CI) became the candidates for the final multivariate logistic regression. Hence, the features included in the final model were age, sex, hemodynamic status, preoperative sepsis, residence, GI contamination, surgical site infection, postoperative cough and/or vomiting, and duration of surgery. Logistic regression analyses were utilized, and a *P*-value < 0.05 was considered statistically significant with a confidence interval (CI) of 95%.

### Ethical considerations

The Institutional Health Research and Ethics Review Committee (IHRERC) at Wolaita Sodo University's College of Health and Medical Sciences granted ethical approval for the study. To obtain administrative approval, a formal letter of collaboration was presented in writing to the WSUCSH before the start of data collection. The hospital and department chiefs gave their informed, voluntary consent after being made aware of the goals, objectives, and advantages of the study. Throughout the process of gathering data and disseminating information, confidentiality of information was upheld.

## Result

This study included a total of 309 patients. Out of these, 198 (64.1%) were male patients and 35.9% female. The age of the participants was >42 years old for 156 patients (50.5%), and the type of residence was found to be rural for 236 (84.6%) patients among the subjects and/or participants incorporated in the study. Moreover, the evidence indicates that 31 (10%) patients had a history of hospitalization, and 249 (80.6%) patients were managed in public wards, which contrasts with an individual private room per patient with a single bed, as shown in Table 1.

**Table 1:** Socio-demographic and preoperative-related features.

Variables	Categories	Frequencies	Percentages
Age of the patient	<42 years	153	49.5
	>42 Years Old	156	50.5
Sex	Male Female	198 111	64.1 35.9
Residence	Town	69	22.3
	Rural	240	77.7
History of hospitalization	Yes	31	10.0
	No	278	90.0
Ward type		60	19.4
	Public	249	80.6
	<7	7	2.3
Preoperative HGB	7–10	40	12.9
	>10	262	84.8
Comorbidities		24	7.8
	No	285	92.2
	DM	7	29.2
Type of comorbidities	HTN	6	25.0
	HIV	3	12.5
	Cancer	8	
	1.00	68	22.0
ASA score	2.00	232	75.1
	3.00	9	2.9

An SSI affected a total of 90 (29.1%) patients incorporated in the study. For these patients, an SSI developed from a clean wound affected 30/180 (16.6%), from a clean-contaminated wound affected 26/83 (31.3%), a contaminated wound affected 20/30 (66.6%), and a dirty wound affected 14/16 (87.5%). Based on the type of SSI, 80/90 (88.8%) were superficial incisional SSIs, 8/90 (8.8%) deep incisional SSIs, and 2/90 (2.22%) organ/space SSIs. This study incorporated 184 (59.5%) patients from elective surgery and the operation site was abdominal for 219 (70.5%), neck 31 (10%), extremities 20 (6.5%), and the remaining 12.6% was thoracic and other procedures. The type of anesthesia given was spinal for 156 (51.7%) patients, and for the remaining it was general anesthesia, while prophylaxis antibiotics was given for 300 (97.1%) patients; prophylactic antibiotics was given at the induction stage for 200 (67%) patients Table 2). Furthermore, wound class was clean for 180 (58.3%) patients; the total duration of hospital stay was less than or equal to 8 days for 193 (62.4%) patients, and the time of SSI diagnosis was ≤7 days for 60 (66.7%) patients (Table 2).

**Table 2:** Surgery and postoperative related features.

Variables	Categories	Frequencies	Percentages
Type of surgery	Elective	184	59.5
	Emergency	125	40.5
Valid	Abdominal	219	70.9
	Extremities	20	6.5
	Thorax	13	4.2
	Neck	31	10.0
	Other	26	8.4
Duration of surgery	<1 h	35	11.3
	1–2 h	157	50.8
	3–4 h	101	32.7
	>4 h	16	5.2
Anesthesia	General	130	43.0
	Spinal	156	51.7
	Regional	7	2.3
	General & spinal	9	3.0
Prophylactic antibiotics given	Yes	300	97.1
	No	9	2.9
Timing of prophylactic antibiotics	at induction	200	66.6
	30 min before surgery	98	32.6
	30–60 min before surgery	2	0.65
Wound class	Clean	180	58.3
	Clean Contaminated	83	26.9
	Contaminated	30	9.7
	Dirty	16	5.2
Wound care given	Yes	216	69.9
	No	93	30.1
Wound care frequency	Daily	200	92.5
	Twice a day	16	7.5
Drain used	Yes	137	45.4
	No	165	54.6
Hospital stay	≤8 Days	193	62.4
	>8 Days	116	37.6
Surgical site infection	Yes	90	29.1
	No	219	70.9
Type of surgical site infection	Superficial	80	88.8
	Deep	8	8.8
	Organ space	2	2.22
Time of surgical site infection diagnosed	≤7 Days	60	66.7
	>7 Days	30	33.3
Amount of blood loss	<500ml	303	98.1
During surgery	500–1500ml	9	2.9
Drain used	Yes	113	36.6
	No	196	63.4

A bivariate logistic regression was carried out to identify candidate-independent features in this study. Bivariate logistic regression variables with a *P*-value of less than 0.25 were considered candidate features for a final multivariate logistic regression. Thus, the candidate features for the multivariate logistic regression were age, sex, type of surgery, preoperative hemoglobin level, drainage usage, operation site, wound type, presence of comorbidities, history of hospital admission, and duration of surgery and were used to determine the factors for SSI at our study setup. Then, after controlling for confounders, a multivariate logistic regression analysis was done. Belonging to the male sex with an AOR of −4.9; 95% (2.0–11.3), drainage usage with an AOR of −4.46; 95% (1.9–10.3), and abdominal surgery with an AOR of −4.3; 95% (1.3–14.1) were predisposing factors for SSI, while younger female sex, elective surgery, and a duration of surgery of less than 2hr were protective factors for SSI. See Table 3 for associated factors for SSI in our study area.

**Table 3:** Determinants of surgical site infection in the study area.

Variable	Category	B (Coefficient)	Std. Error	Wald	df	Sig. (*p*-value)	Exp(B) [Odds Ratio]	95% CI for Exp(B)
Intercept	-	−0.570	0.837	0.464	1	0.496	-	-
Wound class	1.00 (Clean)	−1.941	0.517	14.092	1	**<0**.**001**	0.144	0.052–0.395
	2.00 (Clean-Cont.)	−1.521	0.525	8.388	1	**0**.**004**	0.219	0.078–0.612
	3.00 (Contaminated)	Reference	-	-	0	-	1.000	-
Duration of Surgery	1.00 (Prolonged)	−1.684	0.395	18.194	1	**<0**.**001**	0.186	0.086–0.402
	2.00 (Normal)	Reference	-	-	0	-	1.000	-
Site of Operation	1.00 (High-risk)	1.460	0.607	5.790	1	**0**.**016**	4.307	1.311–14.149
	2.00 (Low-risk)	Reference	-	-	0	-	1.000	-
Preoperative Hemoglobin	1.00 (Low)	0.868	0.445	3.806	1	0.051	2.381	0.996–5.694
	2.00 (Normal)	Reference	-	-	0	-	1.000	-
Age Group	1 (Older)	−0.567	0.427	1.759	1	0.185	0.567	0.246–1.311
	2 (Younger)	Reference	-	-	0	-	1.000	-
Sex of Patient	1 (Male)	1.589	0.425	13.960	1	**<0**.**001**	4.901	2.129–11.281
	2 (Female)	Reference	-	-	0	-	1.000	-
Residence	1 (Rural)	−0.495	0.460	1.161	1	0.281	0.609	0.248–1.500
	2 (Urban)	Reference	-	-	0	-	1.000	-
Comorbidity	1 (Present)	0.518	0.586	0.783	1	0.376	1.679	0.533–5.292
	2 (Absent)	Reference	-	-	0	-	1.000	-
Type of Surgery	1 (Emergency)	−1.163	0.453	6.585	1	**0**.**010**	0.313	0.129–0.760
	2 (Elective)	Reference	-	-	0	-	1.000	-
Drain Used	1 (Yes)	1.496	0.430	12.113	1	**0**.**001**	4.466	1.923–10.373
	2 (No)	Reference	-	-	0	-	1.000	

* Statistical significance with *p*-value <0.05, and bolded one are less than 0.05 and showing significant association.

## Discussion

This study included a total of 309 patients, of whom 198 (64.1%) were male patients and 39.5% were females. The age of the participants was >42 years old in 156 (50.5%) patients, and the type of residence was found to be rural for 236 (84.6%) patients. In our study group, 24 (7.8%) patients had comorbidities and the commonest of these were malignancy in 8 (33.3%), DM in 7 (29.2%), hypertension in 6 (25%), and others made up the remaining share. Prophylactic antibiotics was given to 300 (97.1%) patients, out of whom 200 (66.6%) consumed them during the induction of anesthesia and 98 (32.6%) took them 30 min before operation. Based on carefully thought-out prospective clinical investigations, the selection of parenteral prophylactic antibiotic medicines, as well as the timing and method of administration, has been standardized. It is typically advised that anesthesia staff give a single intravenous dose of cephalosporin shortly before incision in clean, elective surgical operations involving a foreign body and in clean-contaminated surgeries (2).

The common patient factors that predispose for SSIs found in the study are older age, presence of comorbidities, and existing infection, which are consistent with those of other literature (32). The physiologic factors that predispose for SSIs found in our study are that the preoperative hemoglobin level is less than 10 mg/dl (which was the case for 47(15.2%) patients) and surgical risk factors like prolonged procedure time also had a higher association with SSIs. Independent risk factors like abdominal surgery (219/309 (70.9%) patients, of whom 83/209 (39.7%) developed SSI) and wound class are found to predictors in our study, which is consistent with the literature (32, 33).

The magnitude of SSIs in this study was found to be 29.1It is within the upper range reported in studies conducted in different parts of Sub-Saharan Africa, which varies from 6.8% to 26%. (3). Our study finding is relatively higher than the reports from Rwanda 10.9% (34), Nigeria 27.6% (35), Tanzania 10%–26% (36, 37) and the pooled prevalence of Ethiopia 12.3–25.22% (1, 22). It is also lower than in studies done for Niger 74.9% (38) and Nepal 80% (39) and a rural tertiary hospital in Nigeria 70.1% (40) and Tigrai (75%) (41). These differences may be due to differences in setup and SSI prevention strategies used, method of diagnosis, types of wound class, and inclusion of obstetric procedures.

Our study found that being male is associated with an approximate five times higher likelihood of developing an SSI, with an AOR of 4.9; 95% CI (2.13–11.3), although male sex is seen as a controversial risk factor for SSI. This is consistent with a study done in Germany, where the occurrence of SSI was significantly lower in women, with a rate of 2.92/100, while men developed SSIs in 4.37/100 of cases (42). Another study from Korea showed that being male is an independent risk factor for SSIs, with an AOR 1.67; 95%(1.09–2.58) (43). But there are also other multiple other studies that have reported no correlation between SSIs and gender (13, 44). Drainage tube usage is also associated with SSIs in our study, with an AOR 4.46; 95% CI (1.9–10.3), and this is consistent with studies done in Korea (45), India (46), and Switzerland (47), which stated that the presence of drainage tubes is related to a higher risk for developing SSIs. The presence of drains can serve as a conduit for bacteria, facilitating the entry of microbes into the surgical site (48).

In our study, abdominal surgery was associated with a fourfold higher risk of developing SSI, with an AOR of 4.3 (95% CI: 1.3–14.1). Many studies have shown that abdominal operations involve higher risk in terms of developing an SSI compared with other surgical site locations (49–51). Because of the intricate gastrointestinal tract microbiota, microbial variables play a crucial role in abdominal surgery. Surgery can cause dysbiosis, or an imbalance of microbial communities, which can result in an overabundance of harmful bacteria like Proteobacteria, which includes E. coli, a common culprit in surgical site infections (52). Patients who had surgery for less than two hours had an 81.4% (*p*-value = 0.00) lower chance of developing SSI than those who had surgery for more than two hours. This is consistent with multiple studies (50, 53, 54). This is due to increased microbial exposure in the operating field: longer surgical times would increase the risk of surgical wound contamination (55) and, because of the prolonged surgical process and significant blood loss that leads to tissue hypoxia, it also increases the extent of tissue trauma. An estimate would be that infection rate nearly doubles with each hour of surgery. Moreover, guidelines advised reducing the duration of surgical procedures because, the longer the incision is left open, the greater the chance is that bacteria may enter the surgical site (56).

Our findings showed a 68.7% (*p*-value _0.01) lower risk of surgical site infection for those who underwent elective surgery. This is consistent with most studies (57–59). This is due to inadequate preoperative preparation, lack of proper control of other medical comorbidities, and higher risks for contamination in emergency surgeries. In our study, compared to clean wounds, which were considered protective, contaminated/dirty wounds were 78.1% less likely (p-value: 0.04) and clean-contaminated wounds were 85.6% less likely (p-value: 0.00) to acquire SSI. The risk of surgical site infection increases as the percentage of contamination rises. This is consistent with literature from different studies all over the world (35, 60–62). A trend toward higher rates of postoperative SSIs was observed when progressing from clean to dirty wound procedures. Greater contamination during surgery is indicated by a higher wound class, which means that there are more bacteria in the wound area, the surgeons are more likely to use drainage, and a stoma is likely if abdominal surgery is being carried out. This greatly increases the risk of surgical site infection.

The presence of comorbidity had no significant association with SSIs in our study finding. But studies conducted in Tanzania (19), East and West Gojjam hospitals (24), and systemic reviews and meta-analyses done at the national level in Ethiopia (1) have shown significant associations with SSIs. For example, uncontrolled hyperglycemia impairs leukocyte function and lowers immunity, rendering them unable to fight off invasive microbes. Even innocuous bacteria have the ability to spread swiftly and cause major health problems (63, 64). Hyperglycemia, especially from stress, has been linked to an increased risk of SSI, according to studies. Therefore, research suggested that perioperative blood sugar levels should be less than 200 mg/dl and HbA1C < 8% in order to maximize treatment for patients with diabetes mellitus and lower the risk of complications (3, 56). Patients with preoperative HGB ≤10 mg/dl had no significant association with surgical site infection in our study, although studies conducted in Uganda (20) and Nepal (65) showed that low hemoglobin predisposes a patient for SSIs. This is because low hemoglobin concentrations hinder tissue repair and cause tissue hypoxia, which increases the risk of SSIs. Previous history of hospitalization was not significantly associated with surgical site infection in our study. But other studies, such as reports from and meta-analyses done in Ethiopia, showed patients with previous hospitalization were significantly associated with SSIs (1, 66). This could be because the probability of infection is increased by previous exposure to resistant germs (57, 58). These differences may be due to sample differences, set up, or statistical issues.

## Conclusions and recommendations

The magnitude of SSIs in this study was found to be 29.1%, which is very high. Overall drainage usage, abdominal surgery, and male sex are significantly associated with surgical site infection. While drains can be beneficial in certain contexts, their use should be carefully considered, weighing the potential benefits against the increased risk of SSIs. The decision to use drains should be individualized based on the patient's risk factors and the specific surgical context, as it has high association with surgical site infection. Optimization of patient, anticipating infection and acting accordingly either by prophylaxis or therapeutic antibiotics for wounds based on classes, and taking measures which reduce surgical site infection for abdominal surgery is recommended.

### Limitations of the study

This study had some limitations. First, a retrospective document review was used, which may miss some variables and lacks detailed explanations. Also, due to the nature of the retrospective study design used, it was not easy to establish the cause-effect relationship between the study variables and to make other statistical inferences. The type of drainage used, the number of drainage tubes, and the duration of drainage *in situ* was not detailed in our study due to a lack of full documentations.

## Data Availability

The original contributions presented in the study are included in the article/Supplementary Material; further inquiries can be directed to the corresponding author.

## References

[1] Shiferaw WS Aynalem YA Akalu TY Petrucka PM. Surgical site infection and its associated factors in Ethiopia: a systematic review and meta-analysis. BMC Surg. (2020) 20(1):020–00764. 10.1186/s12893-020-00764-1 PMC7236319 32423397

[2] Pinchera B Buonomo RA Moriello NS Scotto R Villari R Gentile I Update on the management of surgical site infections. Antibiotics. (2022) 11(11). 10.3390/antibiotics11111608 36421250 PMC9686970

[3] Ling ML Apisarnthanarak A Abbas A Morikane K Lee KY Warrier A APSIC Guidelines for the prevention of surgical site infections. Antimicrob Resist Infect Control. (2019) 8:1–8. 10.1186/s13756-019-0638-8 31749962 PMC6852795

[4] Bagheri Nejad S Allegranzi B Syed SB Ellis B Pittet D. Health-care-associated infection in Africa: a systematic review. Bull World Health Organ. (2011) 89(10):757–65. 10.2471/BLT.11.088179 22084514 PMC3209981

[5] Amoran O Sogebi A Fatugase O. Rates and risk factors associated with surgical site infections in a tertiary care center in south-western Nigeria. Int J Trop Dis Health. (2013) 3(1):25–36. 10.9734/IJTDH/2013/1769

[6] Organization W.H. Preventing surgical site infections: implementation approaches for evidence- based recommendations. 2018.

[7] Berrios-Torres SI Umscheid CA Bratzler DW Leas B Stone EC Kelz RR Centers for disease control and prevention guideline for the prevention of surgical site infection, 2017. JAMA Surg. (2017) 152(8):784–91. 10.1001/jamasurg.2017.0904 28467526

[8] O'Hara LM Thom KA Preas MA. Update to the centers for disease control and prevention and the healthcare infection control practices advisory committee guideline for the prevention of surgical site infection (2017): a summary, review, and strategies for implementation. Am J Infect Control. (2018) 46(6):602–9. 10.1016/j.ajic.2018.01.018 29525367

[9] Monahan M Jowett S Pinkney T Brocklehurst P Morton DG Abdali Z Surgical site infection and costs in low- and middle-income countries: a systematic review of the economic burden. PLoS One. (2020) 15(6):1–21. 10.1371/journal.pone.0232960 PMC7272045 32497086

[10] Singh S Jowett S Pinkney T Brocklehurst P Morton DG Abdali Z Surgical site infection rates in six cities of India: findings of the international nosocomial infection control consortium (INICC). Int Health. (2015) 7(5):354–9. 10.1093/inthealth/ihu089 25487724

[11] GlobalSurg Collaborative. Laparoscopy in management of appendicitis in high-, middle-, and low-income countries: a multicenter, prospective, cohort study. Surg Endosc. (2018) 32:3450–66. 10.1007/s00464-018-6064-9 29623470 PMC6061087

[12] Leaper DJ van Goor H Reilly J Petrosillo N Geiss HK Torres AJ Surgical site infection—a European perspective of incidence and economic burden. Int Wound J. (2004) 1(4):247–73. 10.1111/j.1742-4801.2004.00067.x 16722874 PMC7951634

[13] Zwicky SN Jung W-S Lim L Yoo HK Ju J-W Lee H-J No impact of sex on surgical site infections in abdominal surgery: a multi- center study. Langenbecks Arch Surg. (2022) 407(8):3763–9. 10.1007/s00423-022-02691-6 36214869 PMC9722878

[14] Ngaroua Ngah JE Bénet T Djibrilla Y. [Incidence of surgical site infections in sub-saharan Africa: systematic review and meta-analysis]. Pan Afr Med J. (2016) 24:171. 10.11604/pamj.2016.24.171.9754 27795768 PMC5072885

[15] Atif ML Nalwadda CK Buregyeya E Gitta SN Anguzu P Nuwaha F [Evolution of nosocomial infection prevalence in an Algeria university hospital (2001 to 2005)]. Med Mal Infect. (2006) 36(8):423–8. 10.1016/j.medmal.2006.05.002 16876975

[16] Pinkney TD Calvert M Bartlett DC Gheorghe A Redman V Dowswell G Impact of wound edge protection devices on surgical site infection after laparotomy: multicentre randomised controlled trial (ROSSINI trial). Br Med J. (2013) 347:f4305. 10.1136/bmj.f4305 23903454 PMC3805488

[17] Brown S Kurtsikashvili G Alonso-Echanove J Ghadua M Ahmeteli L Bochoidze T Prevalence and predictors of surgical site infection in tbilisi, republic of Georgia. J Hosp Infect. (2007) 66(2):160–6. 10.1016/j.jhin.2007.03.007 17513010

[18] Kibwana UO Manyahi J Sensa V Yongolo SC Lyamuya E. Predictors of surgical site infections among patients undergoing open urological surgery at a tertiary hospital, Tanzania: a cross sectional study. East Afr Health Res J. (2022) 6(1):113–8. 10.24248/eahrj.v6i1.686 36424947 PMC9639641

[19] Mawalla B Manyahi J Sensa V Yongolo SC Lyamuya E. Predictors of surgical site infections among patients undergoing major surgery at bugando medical centre in Northwestern Tanzania. BMC Surg. (2011) 11(21):1471–2482. 10.1186/1471-2482-11-21 PMC3175437 21880145

[20] Lubega A Joel B Justina Lucy N. Incidence and etiology of surgical site infections among emergency postoperative patients in Mbarara Regional Referral Hospital, South Western Uganda. Surg Res Pract. (2017) 6365172(10):12.. 10.1155/2017/6365172 PMC5266862 28168215

[21] Dalhatu A Olaogun A Olaogun A Olayinka AT Ahmed S Timothy G Incidence of surgical site infections (SSIs) among patients undergoing Major surgery at general hospital Funtua, Katsina State. Nigeria. IOSR J Nurs Health Sci. (2014) 3(3):16–21. 10.9790/1959-03311621

[22] Birhanu Y Endalamaw A. Surgical site infection and pathogens in Ethiopia: a systematic review and meta-analysis. Patient Saf Surg. (2020) 14(7):020–00232. 10.1186/s13037-020-00232-y PMC7035652 32110246

[23] Mulu W Kibru G Beyene G Damtie M. Associated risk factors for postoperative nosocomial infections among patients admitted at Felege Hiwot Referral Hospital, Bahir Dar, Northwest Ethiopia. Clin Med Res. (2013) 2(6):140–7. 10.11648/j.cmr.20130206.15

[24] Afenigus AD Shbabawu AT Melese TG. Surgical site infection and associated factors among adult patients admitted in west and east gojjam zone hospitals, Amhara region Ethiopia. Ethiopia Nurse Care Open Acces J. (2019) 6(3):107–12. 10.15406/ncoaj.2019.06.00192

[25] Anderson DJ Podgorny K Berríos-Torres SI Bratzler DW Dellinger EP Greene L Strategies to prevent surgical site infections in acute care hospitals: 2014 update. Infect Control Hosp Epidemiol. (2014) 35(6):605–27. 10.1086/676022 24799638 PMC4267723

[26] Neumayer L Hosokawa P Itani K El-Tamer M Henderson WG Khuri SF. Multivariable predictors of postoperative surgical site infection after general and vascular surgery: results from the patient safety in surgery study. J Am Coll Surg. (2007) 204(6):1178–87. 10.1016/j.jamcollsurg.2007.03.022 17544076

[27] NWOSE, P.C. A Prospective Study of the Incidence of Surgical Wound Infection at the Nnamdi Azikiwe University Teaching Hospital, NNEWI. Nigeria: Faculty of Surgery (2006).

[28] Weldu MG Berhane H Berhe N Haile K Sibhatu Y Gidey T Magnitude and determinant factors of surgical site infection in Suhul hospital Tigrai, northern Ethiopia: a cross-sectional study. Surg Infect (Larchmt). (2018) 19(7):684–90. 10.1089/sur.2017.312 30124378

[29] Yaouba D Ngaroua Ngah JE Perpoint T Amvene JMbo Vanhems P Incidence and risk factors for surgical site infections in N'Gaoundéré regional hospital, Cameroon. Am J Infect Control. (2016) 44(10):1195–6. 10.1016/j.ajic.2016.04.250 27544794

[30] Legesse Laloto T Hiko Gemeda D Abdella SH. Incidence and predictors of surgical site infection in Ethiopia: prospective cohort. BMC Infect Dis. (2017) 17:1–9. 10.1186/s12879-016-2167-x 28158998 PMC5290629

[31] Hartmann M Jönsson K Zederfeldt B. Effect of tissue perfusion and oxygenation on accumulation of collagen in healing wounds. Randomized study in patients after major abdominal operations. Eur J Surg. (1992) 158(10):521–6. 1360822

[32] Cheadle WG. Risk factors for surgical site infection. Surg Infect. (2006) 7(1):s1–7. 10.1089/sur.2006.7.s1-7 16834549

[33] Alemayehu MA Azene AG Mihretie KM. Time to development of surgical site infection and its predictors among general surgery patients admitted at specialized hospitals in amhara region, northwest Ethiopia: a prospective follow-up study. BMC Infect Dis. (2023) 23(1):023–08301. 10.1186/s12879-023-08301-0 PMC10193810 37198551

[34] Mukagendaneza MJ Munyaneza E Muhawenayo E Nyirasebura D Abahuje E Nyirigira J Incidence, root causes, and outcomes of surgical site infections in a tertiary care hospital in Rwanda: a prospective observational cohort study. Patient Saf Surg. (2019) 13(10):019–0190. 10.1186/s13037-019-0190-8 PMC6378727 30820247

[35] Olowo-Okere A Ibrahim YKE Sani AS Olayinka BO. Occurrence of surgical site infections at a tertiary healthcare facility in Abuja, Nigeria: a prospective observational study. Med Sci. (2018) 6(3):60. 10.3390/medsci6030060. PMC6163208 30061516

[36] Bayardorj D Promsatit P Chirangi BM Mahmoud E. Surgical site infections at Shirati KMT Hospital in Northeastern Tanzania. Cureus. (2023) 15(2):e34573. 10.7759/cureus.34573. 36874320 PMC9981550

[37] Fehr J Hatz C Soka I Kibatala P Urassa H Smith T Risk factors for surgical site infection in a Tanzanian district hospital: a challenge for the traditional national nosocomial infections surveillance system index. Infect Control Hosp Epidemiol. (2006) 27(12):1401–4. 10.1086/509855 17152042

[38] Odedina E Eletta EA Balogun RA Idowu O. Isolates from wound infections at federal medical centre, bida. Afr J Clin Exper Microbiol. (2008) 9(1):26–32. 10.4314/ajcem.v9i1.7479

[39] Raza MS Chander A Ranabhat A. Antimicrobial susceptibility patterns of the bacterial isolates in post-operative wound infections in a tertiary care hospital, Kathmandu, Nepal. Open J Med Microbiol. (2013) 3(3):159–163. 10.4236/ojmm.2013.33024

[40] Isibor JO Oseni A Eyaufe A Osagie R Turay A. Incidence of aerobic bacteria and Candida albicans in post-operative wound infections. Afr J Microbiol Res. (2008) 2(11):288–91. Available online at: http://www.academicjournals.org/ajmr

[41] Mengesha RE Kasa BG-S Saravanan M Berhe DF Wasihun AG. Aerobic bacteria in post surgical wound infections and pattern of their antimicrobial susceptibility in Ayder Teaching and Referral Hospital, Mekelle, Ethiopia. BMC Res Notes. (2014) 7:1–6. 10.1186/1756-0500-7-575 25164127 PMC4158133

[42] Langelotz C Mueller-Rau C Terziyski S Rau B Krannich A Gastmeier P Gender-Specific differences in surgical site infections: an analysis of 438,050 surgical procedures from the German national nosocomial infections surveillance system. Viszeralmedizin. (2014) 30(2):114–7. 10.1159/000362100 26288585 PMC4513817

[43] Kim ES Kim HB Song K-H Kim YK Kim H-H Jin HY Prospective nationwide surveillance of surgical site infections after gastric surgery and risk factor analysis in the Korean nosocomial infections surveillance system (KONIS). Infect Control Hosp Epidemiol. (2012) 33(6):572–80. 10.1086/665728 22561712

[44] Aghdassi SJS Schroder C Gastmeier P. Gender-related risk factors for surgical site infections. Results from 10 years of surveillance in Germany. Antimicrob Resist Infect Control. (2019) 8:95. 10.1186/s13756-019-0547-x 31171966 PMC6547551

[45] Lee HM Park JW Na YC. Influence of drain characteristics and other known risk factors on surgical site infection occurrence in plastic surgery patients. J Wound Manag Res. (2023) 19(1):28–37. 10.22467/jwmr.2022.02341

[46] Mujagic E Zeindler J Coslovsky M Hoffmann H Soysal SD Mechera R The association of surgical drains with surgical site infections – a prospective observational study. Am J Surg. 217(1):17–23. 10.1016/j.amjsurg.2018.06.015 29935905

[47] Mujagic E Zeindler J Coslovsky M Hoffmann H Soysal SD Mechera R The association of surgical drains with surgical site infections—a prospective observational study. Am J Surg. (2019) 217(1):17–23. 10.1016/j.amjsurg.2018.06.015 29935905

[48] Barbadoro P Marmorale C Recanatini C Mazzarini G Pellegrini I D'Errico MM May the drain be a way in for microbes in surgical infections? Am J Infect Control. (2016) 44(3):283–8. 10.1016/j.ajic.2015.10.012 26717874

[49] Alkaaki A Al-Radi OO Khoja A Alnawawi A Alnawawi A Maghrabi A Surgical site infection following abdominal surgery: a prospective cohort study. Can J Surg. (2019) 62(2):111–7. 10.1503/cjs.004818 30907567 PMC6440888

[50] Zabaglo M Leslie SW Sharman T. Postoperative wound infections. In: StatPearls [Internet]. Treasure Island (FL): StatPearls Publishing (2025). Available from: https://www.ncbi.nlm.nih.gov/books/NBK560533/ (Accessed March 05, 2024). 32809368

[51] Azoury SC Farrow N Hu Q Soares K Hicks C Azar F Postoperative abdominal wound infection—epidemiology, risk factors, identification, and management. Chron Wound Care Manag Res. (2015) 2015(2):137–48. 10.2147/CWCMR.S62514

[52] Spari D Zwicky SN Yilmaz B Salm L Candinas D Beldi G. Intestinal dysbiosis as an intraoperative predictor of septic complications: evidence from human surgical cohorts and preclinical models of peritoneal sepsis. Sci Rep. (2023) 13(1):023–49034. 10.1038/s41598-023-49034-z PMC10739899 38129468

[53] Cheng H Chen BP-H Soleas IM Ferko NC Cameron CG Hinoul P. Prolonged operative duration increases risk of surgical site infections: a systematic review. Surg Infect. (2017) 18(6):722–35. 10.1089/sur.2017.089 PMC5685201 28832271

[54] Scigliano NM Carender CN Glass NA Deberg J Bedard NA. Operative time and risk of surgical site infection and periprosthetic joint infection: a systematic review and meta-analysis. Iowa Orthop J. (2022) 42(1):155–61. 35821941 PMC9210401

[55] Thanni LO Aigoro NO. Surgical site infection complicating internal fixation of fractures: incidence and risk factors. J Natl Med Assoc. (2004) 96(8):1070. 15303412 PMC2568501

[56] Curless M Infection prevention and control: reference manual for health care facilities with limited resources. Baltimore, MD. USA: Jhpiego (2018).

[57] Suchitra Joyce B Lakshmidevi N. Surgical site infections: assessing risk factors, outcomes and antimicrobial sensitivity patterns. Afr J Microbiol Res. (2009) 3(4):175–9. Available online at: http://www.academicjournals.org/ajmr

[58] Mathur P. Hand hygiene: back to the basics of infection control. Indian J Med Res. (2011) 134(5):611–20. 10.4103/0971-5916.90985 22199099 PMC3249958

[59] Misha G Chelkeba L Melaku T. Incidence, risk factors and outcomes of surgical site infections among patients admitted to Jimma Medical Center, South West Ethiopia: prospective cohort study. Ann Med Surg. (2021) 65(102247). 10.1016/j.amsu.2021.102247 PMC8058519 33898031

[60] Ortega G Rhee DS Papandria DJ Yang J Ibrahim AM Shore AD An evaluation of surgical site infections by wound classification system using the ACS-NSQIP. J Surg Res. (2012) 174(1):33–8. 10.1016/j.jss.2011.05.056 21962737

[61] Mioton LM Jordan SW Hanwright PJ Bilimoria KY Kim JY. The relationship between preoperative wound classification and postoperative infection: a multi-institutional analysis of 15,289 patients. Arch Plast Surg. (2013) 40(5):522–9. 10.5999/aps.2013.40.5.522 24086804 PMC3785584

[62] Lilani SP Jangale N Chowdhary A Daver GB. Surgical site infection in clean and clean-contaminated cases. Indian J Med Microbiol. (2005) 23(4):249–52. 10.1016/S0255-0857(21)02530-5 16327121

[63] Nwankwo EO Ibeh I Enabulele O. Incidence and risk factors of surgical site infection in a tertiary health institution in Kano, Northwestern Nigeria. Int J Infect Control. (2012) 8(4). 10.3396/ijic.v8i4.035.12

[64] Martin ET Kaye KS Knott C Nguyen H Santarossa M Evans R Diabetes and risk of surgical site infection: a systematic review and meta- analysis. Infect Control Hospital Epidemiol. (2016) 37(1):88–99. 10.1017/ice.2015.249 PMC4914132 26503187

[65] Giri S Kandel BP Pant S Lakhey PJ Singh YP Vaidya P. Risk factors for surgical site infections in abdominal surgery: a study in Nepal. Surg Infect (Larchmt). (2013) 14(3):313–8. 10.1089/sur.2012.108 23672239

[66] Awoke N Arba A Girma A. Magnitude of surgical site infection and its associated factors among patients who underwent a surgical procedure at Wolaita Sodo University Teaching and Referral Hospital, South Ethiopia. PLoS One. (2019) 14(12). 10.1371/journal.pone.0226140 31805161 PMC6894841

