# Correction: The burden of surgical site infection and associated factors among patients admitted to the surgical ward in resource-limited countries: an institutional-based cross-sectional study

**DOI:** 10.3389/fsurg.2025.1697172

**Published:** 2025-10-09

**Authors:** Simachew Zewdu, Abel Daniel, Amene Abebe, Zinabu Abraham, Hailu Elias, Adamu Belete

**Affiliations:** 1 Department of Surgery, School of Medicine, Wolaita Sodo University, Sodo, Ethiopia; 2 School of Public Health, Wolaita Sodo University, Sodo, Ethiopia; 3 Department of Orthopedic and Trauma Surgery, Gondar University, Gondar, Ethiopia

**Keywords:** surgical site infection, surgical ward, burden, resource-limited setup, factors

Affiliation 1 **Department of Surgery, School of Medicine, Wolaita Sodo University, Ethiopia** was erroneously given as 1Department of Nursing, College of Health Science, Debre Berhan University, Debre Berhan, Ethiopia.

Affiliation **2 School of Public Health, Wolaita Sodo University, Ethiopia** was erroneously given as 2 Department of Clinical Medicine and Surgery—Section of Infectious Diseases, University of Naples “Federico II”, Via Sergio Pansini, Naples Berhan, Italy.

Affiliation **3 Department of Orthopedic and Trauma Surgery, Gondar University, Gondar, Ethiopia** was erroneously given as 3 Department of Orthopedic and Trauma Surgery, Gondar, Ethiopia.

The original version of this article has been updated.

